# Before the Lords' Committee

**Published:** 1890-06-28

**Authors:** 


### Before the Lords' Committee.

(By Our Special Commissioner.)

### V.—SIR SYDNEY H. WATERLOW, BART.

### St. Bartholomew's Hospital.

Sir Sydney said he had been treasurer of St. Bartholomew's Hospital since 1874, and during the last sixteen years the alterations made had been so considerable that the hospital was not the same place as formerly. The whole of the medical school had been rebuilt at a cost of more than £100,000; a Convalescent Home, originally given by Sir Sydney, and situated at Highgate, with 32 beds, was a few years ago removed to Swanley and now has 70 beds. This home differs from nearly all others, as it has skilled trained nurses, a medical man in attendance, and cases are received which require medical treatment in order to relieve the pressure on the beds at Bartholomew's. Each patient remains from nineteen to twenty-six days or longer, at the discretion of the medical officer, who is a practitioner in the neighbourhood living next door to the Home. Last year 165,126 persons received treatment at St. Bartholomew's, of which 6,997 were in-patients, 19,001 were out-patients, 137,399 were casualties, and 1,729 were treated in the maternity department. Patients come from all parts of London, and a great many from the home counties. The hospital contains 660 beds, distributed amongst 28 wards, inclusive of 80 cots. There are 345 male and 303 female beds, and of these beds 198 are medical, 366 are surgical, 25 are ophthalmic, 20 are uterine, 16 are for diphtheria, 23 for erysipelas, 15 are casualty, and 4 for isolated cases. There are four senior physicians, five senior surgeons, two ophthalmic surgeons, two obstetric physicians, two senior and two assistant dental surgeons, one aural surgeon, an assistant surgeon in charge of the skin department, an assistant surgeon in charge of the orthopædic cases, an assistant surgeon in charge of throat cases, a physician in charge of the electrical department, and thirteen house surgeons, house physicians, and other medical officers. The senior surgeons and physicians receive 100 guineas, and the assistants £100 a-year each.

### Government of the Hospital.

Bartholomew's is governed by a body of 273 governors. A governor may be elected by the general body of governors, either for special services or on giving a large sum of money. No one can buy a governorship, and no one is elected who does not either give money or render some special service to the hospital. The president, treasurer, and almoners may nominate governors. The governors elect the president and the treasurer, the latter of whom is assisted by four almoners. One almoner retires every year, so that each serves for four years. Two may have served previously, but two must never have served before. The governors meet quarterly, the quorum is thirteen, and the attendance varies from 150 to 30. The committee of almoners meet on Thursdays at eleven. Property can only be dealt with, and appointments made to the senior offices by the court of governors on the recommendation of the House Committee. The House Committee, consisting of the treasurer, the almoners, and from 26 to 28 other governors appointed by the Court, meets once a month, or oftener if required. The minutes of the House Committee are all read at the Court for confirmation. The government is entirely honorary from the treasurer downwards. The treasurer has power to suspend any officer, and those appointed by the almoners may be dismissed by them. If appointed by the House Committee, the House Committee have the power of dismissal; and when an appointment is made by the Court, the power of dismissal is vested in the Court alone. The treasurer has power to call a court or a meeting of the House Committee, or of the almoners at any time at his discretion. There is a Medical Council, consisting of the physicians, the surgeons, the assistant physicians, the assistant surgeons, the physician accoucheur, the assistant physician accoucheur, and of the junior assistant physician or the junior assistant surgeon, as the Medical Council may from time to time determine, the latter acting as secretary. The Medical Council meets quarterly, or at any time if required by the treasurer. Every honorary physician must be a Fellow of the Royal College of Physicians of London, and every honorary surgeon must be a Fellow of the Royal College of Surgeons of England. Sir Sydney stated that this latter restriction created no difficulty through restricting the number of candidates for the distinguished positions of physicians and surgeons to Barts. The annual income of the hospital amounts to £67,000.

### MR. HUGH WOODS, M.D., AGAIN.

This gentleman writes: As The Hospital has already devoted to myself personally an amount of space which my modesty compels me to think more than my present humble position entitles me to, I am the more anxious to rectify an error as to my personal history into which The Hospital of June 14th has inadvertently fallen. I am there reported to have said before the Lords' Committee that I am an Irishman, and that I have been in this country three years. The writer of the report appears to have been "romancing" with the aid of the "Medical Directory"; for I certainly did not pervert the truth in the manner represented. The same report says that my evidence afforded much amusing reading to the "initiated"; but as the reporter evidently neither read nor heard my evidence, he is obviously not one of the "initiated" alluded to, and must be speaking from information of a very untrustworthy character. I regret that The Hospital thinks our onslaught "very small potatoes." Perhaps we are too sparing in our denunciations of the wolves in sheeps' clothing who are devouring the heritage of the poor. As The Hospital suggests, we must do better in future.

[Are we to understand that Mr. Hugh Woods now denies that he informed the Lords' Committee that he was "an Irish Medical Practitioner," *i.e.*, that he studied medicine entirely in Ireland, and holds an Irish qualification, having been "educated in Dublin"? If he does, the Lords' Committee will know what to do; if he does not, where is the point in the above letter, as we merely stated the facts given in inverted commas? Does he further deny that the chairman pressed him as to his practical experience, or that he pointed out that Mr. Woods was "making use of what is called second-hand evidence"? If he cannot deny this, and of course he cannot, then we repeat that his evidence affords much amusing reading to the initiated, as those who have perused it must admit.—Ed. *T. H.*]

### THE BABY "DREADNOUGHT."

In the most beautiful weather the Prince and Princess of Wales opened the new branch hospital, situated at Canning Town, near the Victoria and Albert Docks, in connexion with the old Dreadnought. Everything from first to last went perfectly, and the whole proceedings reflected the greatest credit upon the committee charged with the arrangements, and the secretary, Mr. Pietro Michelli. Many thousands of people were present. The new hospital was much admired, and should be well-nigh perfect, seeing that it has been built under the direction of Mr. Keith D. Young, the eminent hospital architect. Purses of money were received by the Princess, after which the Prince declared the hospital open, and assented on behalf of the Princess to one of the wards being called the Alexandra. We are glad to know that everybody who took any part in the proceedings felt gratified and pleased, and we hope the new hospital has a long and prosperous career before it. The favours (being the Danish colours with an anchor) worn by the committee, were much admired, the Princess accepting one of them and wearing it —no slight compliment to Mrs. Michelli, who designed and made them.

